# SeesawPred: A Web Application for Predicting Cell-fate Determinants in Cell Differentiation

**DOI:** 10.1038/s41598-018-31688-9

**Published:** 2018-09-06

**Authors:** András Hartmann, Satoshi Okawa, Gaia Zaffaroni, Antonio del Sol

**Affiliations:** 1 Luxembourg Centre for Systems Biomedicine (LCSB), University of Luxembourg, 7. avenue des Hauts-Fourneaux, Esch-sur-Alzette, L-4362 Luxembourg City, Luxembourg; 2 Moscow Institute of Physics and Technology, Dolgoprudny, 141701 Russia

**Keywords:** Computational models, Gene regulatory networks, Software

## Abstract

Cellular differentiation is a complex process where a less specialized cell evolves into a more specialized cell. Despite the increasing research effort, identification of cell-fate determinants (transcription factors (TFs) determining cell fates during differentiation) still remains a challenge, especially when closely related cell types from a common progenitor are considered. Here, we develop SeesawPred, a web application that, based on a gene regulatory network (GRN) model of cell differentiation, can computationally predict cell-fate determinants from transcriptomics data. Unlike previous approaches, it allows the user to upload gene expression data and does not rely on pre-compiled reference data sets, enabling its application to novel differentiation systems. SeesawPred correctly predicted known cell-fate determinants on various cell differentiation examples in both mouse and human, and also performed better compared to state-of-the-art methods. The application is freely available for academic, non-profit use at http://seesaw.lcsb.uni.lu.

## Introduction

Cellular differentiation is a complex biological process, where pluripotent, multipotent or progenitor cells evolve into more specialized cells. Although the detailed mechanism of the underlying process and the pathways involved are not fully understood, it is generally accepted that a few TFs play a crucial role in determining cell fates. The key challenge of computational methods predicting cell-fate determinants from molecular profiling data is how to select a small, but meaningful subset of candidate TFs driving the process. Several valuable data-driven approaches have been proposed for the inference of cell-fate determinants, see for example^1^ and references therein. CellNet is a network biology approach applied to stem cell engineering^2^. The input of the method is gene expression profiles and it predicts similarity to the training samples (basis), the best matching networks from the basis and the importance of the TFs to target for dysregulation. Mogrify^3^ was developed in order to predict combinations of TFs capable of inducing direct conversions between 173 human tissue and cell types. The approach of D’alessio systematically identifies candidate core TFs that represent cell-type identity. It was also demonstrated that these predictions can identify factors capable of converting cell identity. Another data-driven approach was implemented by Heinäniemi *et al*.^4^ to identify putative determinants of cell fate based on the concept of gene expression reversal of gene pairs, such as those participating in toggle-switch circuits.

However, as shown in the comparative summary of the methods (Table 1), current tools require a comprehensive cell/tissue library as training- or background data sets, preventing users from inputting new transcriptomics data and not all of them provide publicly accessible source code. To the best of our knowledge, no general tool exists for the identification of candidate cell-fate determinants for any given differentiation event, even though the importance of such tools is evident in cell engineering, regenerative medicine and cancer therapeutics^5,6^. Here we developed SeesawPred, a publicly available web-based application framework that can systematically predict cell-fate determinants based on a GRN model of cell differentiation. As it allows the users to upload their own transcriptomics data and does not rely on pre-compiled reference data sets, it is well-suited for identifying cell-fate determinants in novel differentiation systems.

**Table 1:** Comparison of major properties between different methods for predicting cell-fate determinants.

	SeesawPred	Mogrify	D’Alessio	CellNet
GRN requirement	Yes	Yes	No	Yes
Cell/tissue library requirement	No	Yes	Yes	Yes
Accepts user input data	Yes	No	No	Yes/No*
Model of differentiation	Yes	No	No	No
Source availability	Yes	No	No	Yes

*Only starting cell/tissue type.

## Methods

Here we extended the framework introduced by^7^ and built a web-based application. Conceptually, the method is based on a GRN model, where cell-fate determinants (lineage specifying TFs) reside in a general class of a network motif, strongly connected component (SCC) and govern the gene expression programme that stabilizes three phenotypes (stem/progenitor cell type and two daughter cell types from the stem/progenitor cell). Moreover, opposing cell-fate determinants are assumed to be differentially expressed between the two daughter cell types, but in the stem/progenitor cell they show a balanced gene expression pattern. This assumption also corresponds to the “*seesaw*” model of stem cell differentiation^8–10^ and can be formally expressed with the measure of normalized ratio difference (NRD)^7^, for each TF pair *C*_*i*_, *C*_*j*_, *i* ≠ *j* as

1 $$NR{D}_{i,j}=\frac{\frac{{C}_{i}^{P}}{{C}_{j}^{P}}-\frac{{C}_{i}^{D}}{{C}_{j}^{D}}}{\frac{{C}_{i}^{P}}{{C}_{j}^{P}}},$$

where *C* represents the gene expression and the upper index shows if the TF *i* and *j* are from the Stem/Progenitor (*P*) or Daughter (*D*), respectively.

**Figure 1:**
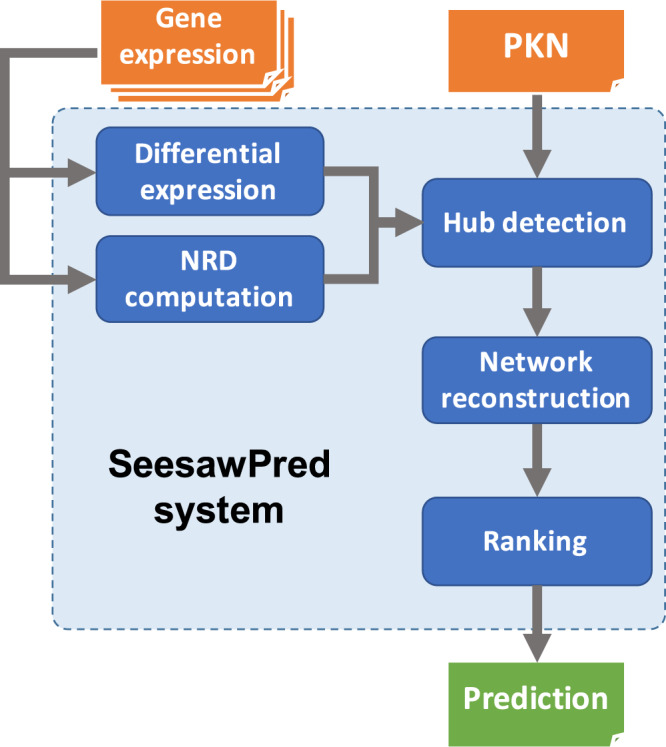
Schematic pipeline of the SeesawPred application.

The building blocks of the application are shown in Fig. 1. The input consists of the gene expression profiles of the stem/progenitor and two daughter cell types and a prior knowledge network (PKN). In case a PKN is not readily available, the application also includes built-in human and mouse general-purpose transcriptional regulatory networks retrieved from MetaCore^11^ on 02/26/2018 (see Supplementary Table S3), which can be used for limited-size applications (<1000 records). All these interactions are reported in literature and no predicted interactions are included. First, differentially expressed TFs between the two daughter cell types are derived and the NRD is calculated for each daughter cell type and for each TF pair. The NRD pairs are then tested for significance and filtered. The criteria for keeping a TF are that it has to:

1. be differentially expressed between the two daughter cell types,
2. constitute significant NRD pairs on significance test among all TFs and
3. show high absolute NRD values in both daughter cell types (|*NRD*| > 0.5).

In the next step, the PKN is subset with only the TFs that meet the above criteria. In addition, the low-degree nodes are filtered out from the network by only keeping the nodes with out-degree larger than six. Although TFs with out-degree less than equal six could potentially be cell-fate determinants, this cutoff is chosen, because all experimentally validated cell-fate determinants we found for the differentiation events used in this study have out-degrees larger than six and setting this cutoff was found to reduce the run time without decreasing the overall performance of the method (see the sensitivity analysis in Supplementary Figure S4). Then, the network is pruned using the Boolean GRN formalism, such that the GRN is a sub-network of the PKN, where the edges do not conflict with the differential gene expression states between the two daughter cell types.

Different GRN solutions are generated, since the differential gene expression states can be explained by topologically different GRNs. The number of the different GRN solutions is a user-defined parameter, with an upper limit of 10.000 networks. Finally, SCCs are detected in each GRN solution. The final score for a TF pair is calculated by multiplying the following three factors:

1. Fraction of SCCs they are present in.
2. Fraction of SCCs where they are directly connected.
3. Minimum out-degree.

### Implementation

SeesawPred was implemented in R statistical language and the web interface has been developed using the Shiny web technology, with all major browsers supported. The algorithm has been optimized in order to provide response time adequate for practical applications. The source code is available under the terms of the Affero GPL license v3 together with the deployed webtool at http://seesaw.lcsb.uni.lu.

The required input of SeesawPred is a tab separated value file where columns represent gene expression (microarray or RNA-Seq) replicates of the progenitor cell type and the two daughter cell types labeled as “Progenitor”, “Daughter1”, “Daughter2”, respectively, and the rows are labeled according to the gene symbols. The user-defined PKN is also a tab separated value file containing the list of TF - TF interactions that serve as potential links in the network. The first and the third columns correspond to source and target TFs, respectively, and the second column describes the interaction type which can be “Activation”, “Inhibition” or “Unspecified”. For this study, we used a literature-based, curated PKN consisting of edges that describe direct interactions of binding, transcriptional regulation and influence on expression as retrieved from MetaCore. However, users can input any interactions of their interest, such as those originated from genome-wide ChIP-seq experiments or computational predictions. Example input files are contained in the help section of the application.

## Results

### Validation of the method

The validity of SeesawPred is tested against various binary cell differentiation examples in both human and mouse, for which transcriptomics data and known cell-fate determinants are available (Table 2). The example data are directly accessible from SeesawPred. The predicted cell-fate determinants in these examples are consistent with literature evidence and the most representative, predicted TF pairs are listed in Table 2. For example, well-known toggle switches, GATA1__SPI1 and PPARG__RUNX2 are predicted for the HSC and MSC differentiation events, respectively. Another example is GATA3__STAT1 that specifies either Th2 or Th1 cells from CD4+ helper T cells. Besides known cell-fate determinants, additional TFs are predicted, which constitute putative, novel cell-fate determinants that can be prioritized for experimental validation.

**Table 2:** Example data set with representative known cell-fate determinants predicted by SeesawPred.

Example	Description	Representative pair
hDE	DE to PF and MG/HG	CDX2__PAX6; SOX2__TBX3
hDE 2	DE to PF and MG/HG	CDX2__SOX2
hHSC	HSC to GMP and MEP	GATA1__SPI1
mCBC	CBC to SP and AP	FOXA1__MYC; KLF4__MYC
mHSC	FDCP-mix to RBC and MYC	GATA1__SPI1
mMEP	MEP to RBC and MK	ETS2__SP1; FOXO3__RUNX2
mMONO	MONO to MDDC and MACRO	CEBPA__IRF8
mMSC	MSC to OST and AD	PPARG__RUNX2
mNSC	NSC to NEU and ASTRO	ESR1__RUNX2
mTHC	Th to Th1 and Th2 cells	GATA3__STAT1

Abbreviations: definitive endoderm (DE), posterior foregut (PF), midgut (MG), hindgut (HG), hematopoietic stem cell (HSC), granulocyte-macrophage progenitor (GMP), megakaryocyte-erythroid progenitor (MEP), crypt base columnar cell (CBC), secretory progenitor (SP), absorptive progenitor (AP), erythrocyte (RBC), myeloid cell (MYC), megakaryocyte (MK), monocyte (MONO), monocyte-derived dendritic cell (MDDC), macrophage (MACRO), mesenchymal stem cell (MSC), osteoblast (OST), adipocyte (AD) neuronal stem cell (NSC), neuron (NEU), astrocyte (ASTRO), T helper cell (Th).

### Comparison with other methods

In order to quantify the performance of SeesawPred, we benchmarked it against three other methods that can predict TFs for cellular transitions: Mogrify^3^, CellNet^2^ and the method of D’Alessio *et al*.^12^. Supplementary Table S2 compares ranked lists of predicted TFs for eight cell differentiation examples, for which predictions from both Mogrify and D’Alessio *et al*. are available. CellNet results are shown wherever the target cell type is available in its built-in data set. In some cases, the exact same cell type is not available and these cases are either completely excluded, or close cell/tissue types are used as surrogates (*e.g*. Mogrify does not contain erythrocytes and myeloid cells and “blood - adult” is used instead and CellNet has only three matching cell types out of eight used cases) (Supplementary Table S2). The comparison is based on how many known (experimentally validated) cell-fate determinants are recovered in the top predictions of each method. Results are trimmed to maximum eight predicted TFs, as Mogrify only reports up to eight predictions. Since Mogrify, CellNet and the method of D’Alessio *et al*. are not designed only for cell differentiation, TFs that are known for cellular reprogramming to respective target cell types are included in the benchmarking. The complete list of used cell-fate determinants with literature evidence is shown in Supplementary Table S1.

**Figure 2:**
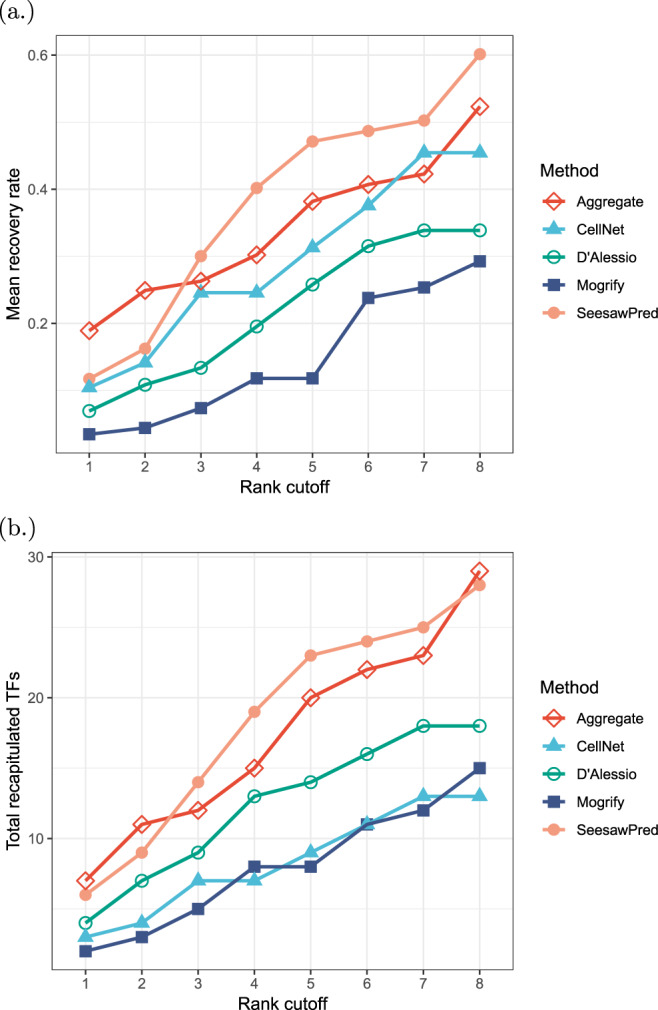
Comparison of different tools in recovering known cell-fate determinants at different rank cutoffs (x-axis) against (**a**) recovery rate calculated by the number of recovered known cell-fate determinants divided by the number of total known cell fate determinants, and (**b**) the number of recovered known cell-fate determinants. Values are based on eight differentiation cases (HSC to erythrocyte, HSC to myeloid cell, monocyte to monocyte-derived dendritic cell, monocyte to macrophage, MSC to osteoblast, MSC to adipocyte, NSC to neuron, and NSC to astrocyte). Since CellNet does not have target cell/tissue types for five cases, recovery rate is calculated based only on the other three cases for (**a**) and the number of recovered known cell-fate determinants is considered zero for these five cases for (**b**). Aggregated rankings are computed from the ranked lists of all the four methods including SeesawPred by reciprocal rank fusion. For more details, see also Supplementary Table S2.

The result indicates that in both average recovery rate and total number of recovered cell-fate determinants SeesawPred performs the best at each rank cutoff in comparison to the other three individual methods (Fig. 2). Although CellNet ranks the 2nd best in the average recovery rate as an individual method (Fig. 2a), this result is based only on three cases, as it does not have the target cell types for the other five cases. For this reason, the performance of CellNet drops when the total number of recovered cell-fate determinants is considered (Fig. 2b) and the method of D’Alessio *et al*. performs the 2nd best as an individual method. Next, we aggregate the predictions from all the four methods, including SeesawPred, to see if this results in an increased performance. To this end, we use the reciprocal rank fusion^13^, that sorts based on the formula:

2 $$RRFscore(d\in D)=\sum _{r\in R}\,\frac{1}{k+r(d)},$$

where *D* is a set of TFs to be ranked based on a set of rankings *R*, which are permutations on 1..|*D*|. The constant *k* = 10 is fixed *a priori*. New top eight TFs are taken based on this new ranking (also in Supplementary Table S2, “Aggregate” rows). This rank aggregation shows a slightly better performance than SeesawPred at some rank cutoffs (“Aggregate” method in Fig. 2), however, it does not result in any significant improvement in the overall performance: an improvement in three cases, one tie, a decrease in performance in four cases.

Note, in some cases the other individual methods perform better than SeesawPred. For example, in the cases of monocyte-to-macrophage and NSC-to-neuron differentiation, the method of D’Alessio *et al*. and CellNet recover more known cell-fate determinants than SeesawPred. In addition, MITF and/or TFEC are predicted by the other methods for macrophage and these TFs are indeed implicated in the macrophage lineage^14–16^. However, we could not find direct evidence that they can induce macrophage differentiation or reprogramming and therefore, they are not counted as known cell-fate determinants. In general, it is not straightforward to directly compare on these broad categories of cell types, since many subtypes are known to exist (*e.g*., different subtypes of neurons and macrophages). Therefore, the used gene expression data likely come from different subtypes among the methods that do not accept user input data. These examples also indicate that no single method performs the best every time and further development of better prediction methods is still necessary.

### Sensitivity analysis

**Figure 3:**
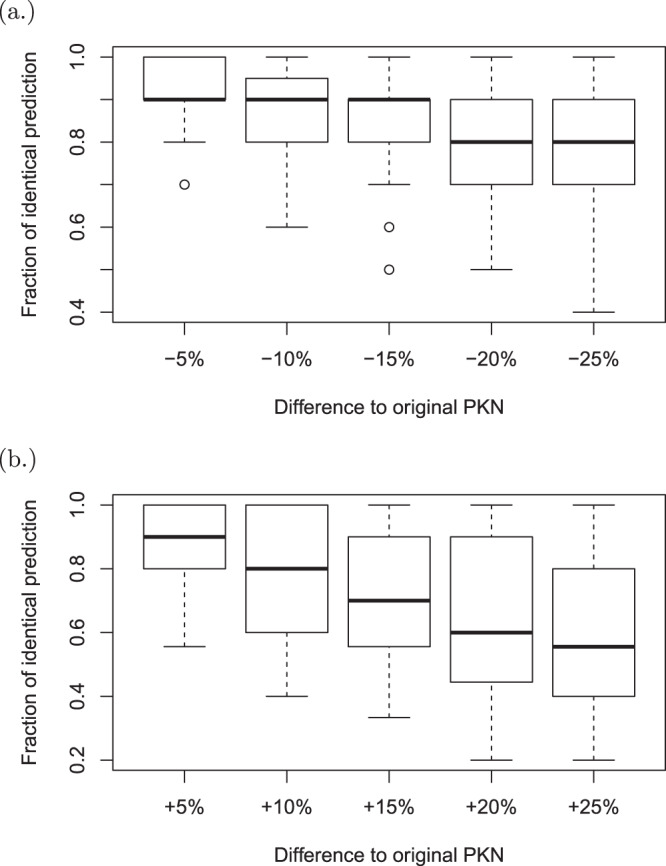
Sensitivity of SeesawPred predictions to randomly removing from (**a**) and adding new edges to the PKN (**b**). The fraction of identical predictions (See Eq. 2) is plotted against the amount of edges removed/added with respect to the size of the original PKN. Each boxplot represents 10 independent trials for each of the eight examples in Supplementary Table S2 (*i.e*., 80 total trials).

Finally, in order to quantitatively assess the robustness of SeesawPred to the PKN, we perturb the original PKN by randomly masking out or adding new regulatory edges and then computing the fraction of top 10 predicted TFs that are also present in the top 10 predictions made from the original PKN. More formally, this fraction is expressed as:

3 $$\frac{|TFs(PKN)\cap TFs(PKN^{\prime} )|}{|TFs(PKN)|},$$

where |·| is the cardinality operator, *TFs*(*x*) represent the TFs predicted with the PKN *x*, the perturbed network is denoted with PKN’. The range of the perturbation is set to ±25% with 5% steps. 10 independent PKNs are generated for each step and each differentiation example contained in Supplementary Table S2, and the fraction of common predicted TFs is computed (Fig. 3). As expected, subsampling or extending the PKN causes some differences in predictions. As the (absolute) percentage of difference between PKNs increases, the median fraction of identical predictions decreases with increasing spread. We observe that this effect is stronger if new edges are added compared to when edges are removed. Nevertheless, the statistics show that removing/adding 5% (≈600) edges maintain the median fraction of 0.9 (90%) of the original SeesawPred predictions. Moreover, even the 25% (≈3000) removal/addition of edges still maintain the median fractions of 0.8/0.5 of the original SeesawPred predictions, respectively.

Given that any GRN-based method is, by construction, supposed to be sensitive to changes in the network topology to a certain degree, SeesawPred seems to be reasonably robust to changes in the PKN. Note, in this sensitivity analysis, for the sake of completeness, we test up to 25% random removals/additions of network edges. However, a (random) change of this extent is unlikely to occur in already comprehensive and well-curated databases.

## Discussion

We present SeesawPred, a web application for predicting cell-fate determinants from transcriptomics data. SeesawPred relies on the assumption that the stem/progenitor cell phenotype is maintained in a metastable state by the opposing cell-fate determinants, which are part of interconnected feedback loops known as SCCs (*e.g*. toggle switches)^7^. During binary cell-fate decisions, the equilibrium is shifted towards either of the two cell-fate determinants and the gene expression profile stabilizes in the corresponding daughter cell type. This is in line with the “*seesaw*” model of cell reprogramming^8–10^ that was proposed to illustrate the balanced equilibrium of the cell-fate determinants in the pluripotent state.

An inherent advantage of SeesawPred is that it can predict cell-fate determinants based on user input transcriptomics data without the need for background or training data set (Table 1). Therefore, it is well-suited for identifying cell-fate determinants in novel differentiation systems, for which no previous data are available. Indeed, among the 18 unique differentiation events we collected (Table 2), only eight of them could be handled by the other methods. As functionally distinct, novel subtypes of cell types have been increasingly identified, we believe SeesawPred will be valuable in guiding differentiation experiments of such novel cell types.

The performance of SeesawPred was also found better than the other existing methods^2,3,12^ in recapitulating known cell-fate determinants based on the eight cell differentiation systems (Fig. 2, Supplementary Table S2). This could be explained by the underlying model of SeesawPred, which is a generalized extension of the experimentally observed GRN model^9,10,17–19^ specifically designed for binary cell differentiation. However, such performance comparisons are not always straightforward, since the other methods are designed more for any type of cell conversions and are not specific for binary cell differentiation. In fact, in the monocyte-to-macrophage and NSC-to-neuron differentiation cases SeesawPred was not the best performing (Supplementary Table S2), possibly because not all cell-fate determinants comply with our cell differentiation model and also possibly because different methods use different gene expression data which may come from different subtypes of broad cell-type categories, such as “neuron” and “macrophage”. In the light of the different predictions from the individual methods, we aggregated the predictions of the four methods, including SeesawPred, using a reciprocal rank fusion. However, this did not result in any significant improvement in the overall performance in comparison to SeesawPred alone (Fig. 2). Thus, development of more sophisticated aggregation methods may be another avenue for better prediction methods.

Finally, the performance comparison was conducted solely on the current knowledge of experimentally validated cell-fate determinants, which is far from complete and makes the accurate calculation of false positive rate impossible. Further experimental efforts to characterize true positive/negative cell-fate determinants are needed for comprehensive performance comparisons. It should also be noted that none of the methods discussed in this study, including SeesawPred, systematically addresses the problem of differentiation efficiency and fidelity. We consider this a general future challenge for the field.

## Conclusion

SeesawPred recapitulated known cell-fate determinants in a number of diverse examples, underscoring the validity of this approach. Moreover, the comparison with already published methods showed a superior performance of SeesawPred. Further validation both computationally and experimentally will reinforce the generalizability of the method. SeesawPred is made publicly available for academic non-profit use in order to guide differentiation experiments in stem cell research and regenerative medicine.

## Electronic supplementary material

Sensitivity

Examples

Benchmark

Network

## Data Availability

All transcriptomics data analysed in this study are publicly available (see Supplementary Table S1 for accession numbers). The PKNs that support the findings of this study are included in the application for reproducibility purposes, and are available from Clarivate Analytics (MetaCore) but restrictions apply to the availability of these data, which were used under license for the current study, and so are not publicly available. Data are however available from the authors upon reasonable request and with permission of Clarivate Analytics.
