# Author Correction: SeesawPred: a web application for predicting cell-fate determinants in cell differentiation

**DOI:** 10.1038/s41598-022-25606-3

**Published:** 2022-12-16

**Authors:** András Hartmann, Satoshi Okawa, Gaia Zaffaroni, Antonio del Sol

**Affiliations:** 1 Luxembourg Centre for Systems Biomedicine (LCSB), University of Luxembourg, 7. avenue des Hauts-Fourneaux, Esch-Sur-Alzette, 4362 Luxembourg City, Luxembourg; 2 Moscow Institute of Physics and Technology, Dolgoprudny, Russia 141701

Correction to: *Scientific Reports* https://doi.org/10.1038/s41598-018-31688-9, published online 06 September 2018

This article contains an error in the Acknowledgements section.

This work was supported by the Fonds National de la Recherche Luxembourg [C15/BM/10397420 to S.O., 10035087 to G.Z.].

should read:

This work was supported by the Fonds National de la Recherche Luxembourg [C15/BM/10397420 to S.O., 10035087 to G.Z., and C17/BM/11662681 to A.H.].

