# Supplementary material for: SeesawPred: A Web Application for Predicting Cell-fate Determinants in Cell Differentiation
Source: Sci Rep. 2018 Sep 6;8:13355. doi: 10.1038/s41598-018-31688-9 (PMC6127256; doi:10.1038/s41598-018-31688-9)
Supplement: Supplementary file 1 — Sensitivity [file 41598_2018_31688_MOESM1_ESM.pdf]

SeesawPred: A Web Application for Predicting Cell-fate Determinants in Cell Differentiation

András Hartmann[1], Satoshi Okawa[1], Gaia Zaffaroni[1], and Antonio del Sol[1,*]

[1]Luxembourg Centre for Systems Biomedicine (LCSB), University of Luxembourg, 7. avenue des Hauts-Fourneaux, Esch-sur-Alzette, L-4362, Luxembourg
[*]Antonio.delSol@uni.lu

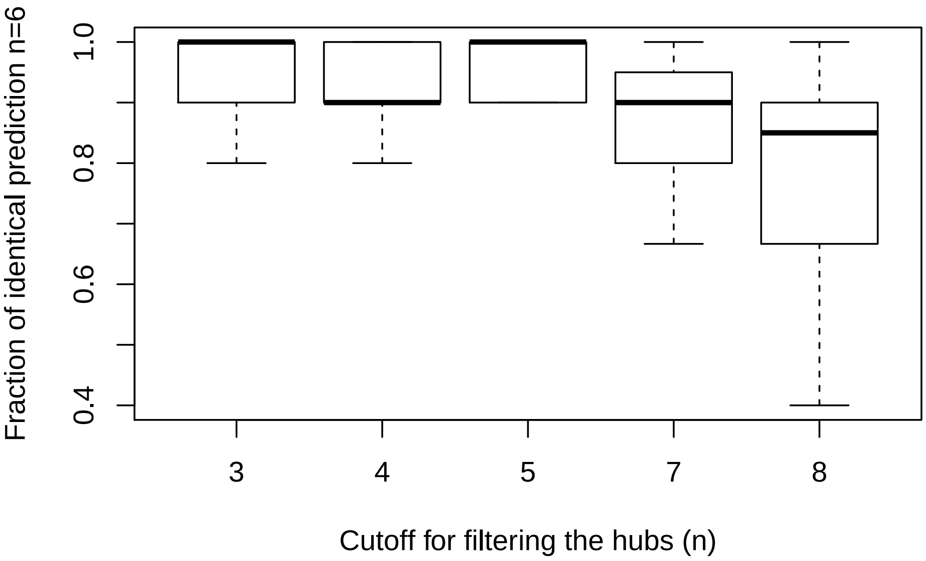

Supplementary Figure S4. Sensitivity to hub filtering cutoff. The sensitivity analysis was performed by comparing the fraction of predicted TFs that were common in the top ten ranking between predictions between the cases when the threshold (n) equals 6 and $n \in \{3,4,5,7,8\}$.
